# Comparative Antimicrobial Activity of Silver Nanoparticles Obtained by Wet Chemical Reduction and Solvothermal Methods

**DOI:** 10.3390/ijms23115982

**Published:** 2022-05-26

**Authors:** Liliana Marinescu, Denisa Ficai, Anton Ficai, Ovidiu Oprea, Adrian Ionut Nicoara, Bogdan Stefan Vasile, Laura Boanta, Alexandru Marin, Ecaterina Andronescu, Alina-Maria Holban

**Affiliations:** 1 Faculty of Chemical Engineering and Biotechnologies, Politehnica University of Bucharest, Gh Polizu Street 1-7, 011061 Bucharest, Romania; liliana.marinescu67@gmail.com (L.M.); denisa.ficai@upb.ro (D.F.); anton.ficai@upb.ro (A.F.); ovidiu73@yahoo.com (O.O.); adi.nicoara18@gmail.com (A.I.N.); bogdan.vasile@upb.ro (B.S.V.); 2 Academy of Romanian Scientists, Ilfov Street 3, 050054 Bucharest, Romania; 3 Department of Hydraulics, Hydraulic Machinery and Environmental Engineering, Faculty of Power Engineering, Politehnica University of Bucharest, 313 Splaiul Independentei, District 6, 060042 Bucharest, Romania; laura.boanta@upb.ro (L.B.); alexandru.marin@upb.ro (A.M.),; 4 Microbiology and Immunology Department, Faculty of Biology, University of Bucharest, 1-3 Portocalelor Lane, District 5, 77206 Bucharest, Romania; alina_m_h@yahoo.com

**Keywords:** antimicrobial activity, silver nanoparticles, synthesis methods, comparative effect

## Abstract

The synthesis of nanoparticles from noble metals has received high attention from researchers due to their unique properties and their wide range of applications. Silver nanoparticles (AgNPs), in particular, show a remarkable inhibitory effect against microorganisms and viruses. Various methods have been developed to obtain AgNPs, however the stability of such nanostructures over time is still challenging. Researchers attempt to obtain particular shapes and sizes in order to tailor AgNPs properties for specific areas, such as biochemistry, biology, agriculture, electronics, medicine, and industry. The aim of this study was to design AgNPs with improved antimicrobial characteristics and stability. Two different wet chemical routes were considered: synthesis being performed (i) reduction method at room temperatures and (ii) solvothermal method at high temperature. Here, we show that the antimicrobial properties of the obtained AgNPs, are influenced by their synthesis route, which impact on the size and shape of the structures. This work analyses and compares the antimicrobial properties of the obtained AgNPs, based on their structure, sizes and morphologies which are influenced, in turn, not only by the type or quantities of precursors used but also by the temperature of the reaction. Generally, AgNPs obtained by solvothermal, at raised temperature, registered better antimicrobial activity as compared to NPs obtained by reduction method at room temperature.

## 1. Introduction

Silver nanoparticles (AgNPs) are currently used in various fields, as medicine [1], biology area as biosensor materials [2], industry as composite fibers [3], textiles, water waste treatment products [4,5], construction area [6], detergents and cosmetic fields [7], electronic area [8] or antimicrobial packaging [9,10,11,12]. The pandemic actual situation brought silver nanoparticles to the attention of researchers with special characteristics regarding the antibacterial and antiviral protection [13,14,15,16]. Noble metal nanoparticles are characterized by a strong plasmon resonance absorbance peaks in the UV-VIS region given by the localized surface plasmon resonance (LSPR) or surface-enhanced Raman scattering (SERS) [17]. Thus, the aspect of obtained nanoparticles, in solution, by chemical methods have various colors depending on the reaction conditions [8,13,18,19,20,21].

AgNPs are well known for possessing an inhibitory effect to the most common microorganisms in the medical and industrial processes [14,22]. AgNPs are leading nanomaterials in the fight against pathogenic microorganisms [14,23,24,25,26]. Comparing to the bulk form of silver, the increased surface area of silver nanoparticles is feature responsible for their special behavior in this regard [8,27,28,29].

There are several methods used in the synthesis of silver nanoparticles such as biological [25,26], physical, chemical, photochemical reduction, and electrochemical, which could ensure an improvement in their antimicrobial properties and physico-chemical properties [30,31]. The morphology and size distribution of nanoparticles have an important influence in their physicochemical properties and these can be controlled during the various synthesis approaches [13,14,32,33].

The chemical reduction method has been used the widest studied, due to the general versatility of the technique [34,35,36]. Chemical reduction is the most common chemical methods used using various reduction precursors which generate the tailored properties of silver nanoparticles [37]. Different techniques are widely optimized methods for synthesis of AgNPs, each presenting advantages and disadvantages. Chemical reduction is the most frequently applied method, being accessible, cost-effectiveness, simpler handling for the preparation of silver nanoparticles as stable, colloidal dispersions in water or organic solvents [38,39].

The advantage of the chemical synthesis of nanoparticles is related to the ease of production, low cost, relatively short time of reaction and high yield [31,40,41]. But, it must be acknowledged that the chemical reduction is considered potentially harmful, with high toxicity comparing with other synthesis routes where precursors are not involved [42,43].

The formation mechanism consists in the reduction of silver ions into free metallic silver atoms which are further lead to the formation of the nanoparticles (by growing), in a bottom–up approach. The chemical reduction consists by the addition of reducing agents as (sodium borohydride, ascorbate, tri-sodium citrate, hydrazine, Tollens reagent, or polyethylene glycol) which lead to form of free metallic silver atoms. As the reaction proceeded, under the influence of reaction conditions, such as magnetic stirring, temperature, pH, these silver atoms accumulated into oligomer clusters and finally these clusters lead to the formation of silver colloids [44,45]. 

The present work proposes two synthesis methods (i) chemical reduction at the room temperature using reducing agents, such as sodium borohydride at different concentrations and tri-sodium citrate. The stabilizing agent polyvynilpirrolidone (PVP) has a role to prevent the agglomeration of nanoparticles during synthesis or deposit them [46]. It was noticed that the PVP capping agent plays an important role in controlling the ratio growth rates for 100 and 111 facets [13,47,48] resulting in the formation of nanocubes and nanorods shapes. Adding the hydrogen peroxide in the last stage of experiment has a role to prevent the oxidation of small particles into Ag^+^ [39,49]. (ii) The chemical reduction at the raised temperature named solvothermal method using polyethylene glycol (PEG) as solvent and capping agent. This route follows the influence of different temperatures (200–260 °C) and reaction time on the silver nanoparticles formation, more precisely shapes, sizes, and consequently the antimicrobial activity. The polyethylene glycol 400 (PEG), with polyvinylpyrrolidone (PVP) are used both as a stabilizing agent [50,51,52,53].

## 2. Materials and Methods

### 2.1. Synthesis by Chemical Reduction at Room Temperature

The chemicals products have analytical grade and were used without further purification. The water used is deionized 18 MΩ. The silver nitrate is from Silal Trading, Bucharest, Romania (99.999%), the sodium borohydride is from Sigma Aldrich Co., Ltd. (Taufkirchen, Germany), the tri-sodium citrate dihydrate is from Merck Co., Ltd. (Darmstadt, Germany) and PVP K30 is from Roth Co., Ltd. (Karlsruhe, Germany). 

**Figure 1:**
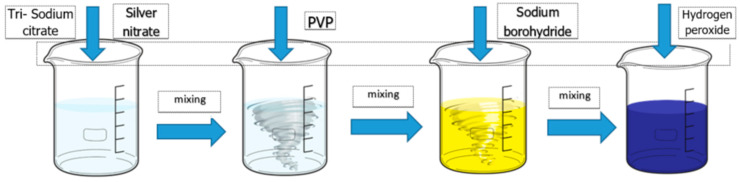
Technological scheme of the chemical routes at the room temperature (RT).

A solution of silver nitrate (0.1 mM) was prepared in 500 mL deionized water in a 1000 mL flask, then was added 30 mL tri-sodium citrate dihydrate (3 × 10^−2^ M) solution mixed vigorously (600–700 rpm) after 10 min was added 30 mL of PVP solution 0.7 mM and various volumes of sodium borohydride. The mixture was magnetic stirring for 2–3 min, at room temperature then added 1–3 mL hydrogen peroxide 35% (Figure 1). The concentration of obtained samples is 10 ppm. The obtained samples were coded with RT1 to RT3 mentioned in the Table 1.

All obtained samples prepared by the chemical reduction at the room temperature, used two type of reducing agents as the trisodium citrate Na_3_C_6_H_5_O_7_ a mild reducing agent and other one a strong reducing agent, sodium borohydride NaBH_4_. The differences between applications are the variable volume added of the strong reducing agent sodium borohydride (NaBH_4_) respectively their molar concentration (Table 1).

### 2.2. Synthesis by Solvothermal Method

**Figure 2:**
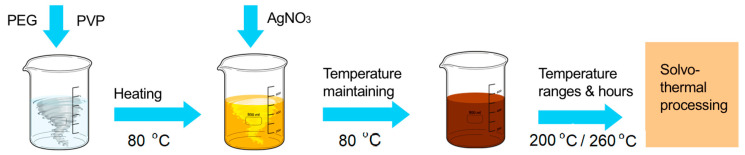
The technology line for the chemical route at the raised temperature (solvothermal synthesis).

The chemical route by solvothermal method uses at the raised temperature with PEG (polyethylene glycol) with an average molecular mass of 400 (Aldrich), analytical purity. Deionized water with resistance of 18 MΩ was used. The synthesis of uniform silver nanospheres using solvothermal way [54] were followed by a polyol method and PEG is the reduction component. The solution was prepared by 888 mg of PVP (K30), added to 80 mL of PEG 400 and stirring at 80 °C until the solution is becoming transparent (Figure 2). The order of addition and quantities of reactants are mentioned in the table (Table 2) Than 2 mL of nitrate silver AgNO_3_ (0.5 M was rapidly injected under the magnetic stirring and heating the mixture (Figure 2). The obtained mixture was poured in a Teflon tank which was introduced in the apparatus for the solvothermal application. The solvothermal reaction takes place at different temperatures (200 °C or 260 °C) maintained for a period time indicated in the table (Table 2) and at constant pressure. The color of solution changed from colorless to light yellow or dark brown colour (Figure 2). The concentration of the obtained samples is 1600 ppm. The obtained samples are coded in this work with HT (high temperature) from 1 to 4 (Table 2)

### 2.3. Characterization of the Obtained Samples 

The UV–Vis spectra were recorded using an Evolution 300 UV-VIS spectrophotometer in absorbance mode (190–1100 nm), 1 nm used as data interval, 2 nm bandwidth and 240 nm/min scan speed, in 10 mm quartz cuvettes, at room temperature. The DLS measurements were done using a DELSA Max Pro, light scattering analyzer reusable PEEK flow cells, BCI-3216-DMP, DLS detector angle (degree) 163.5°. The acquisition parameters for this technique were: acq time (s): 5, read interval(s): 1, number acq: 3, electric field frequency (Hz): 10, collection period (s): 15, auto-attenuation: yes, attenuation level (%): 0, laser mode: normal, set temp on connection: no, set temp (°C): 20. The dilution factor used was 10 (1 mL in 10 mL of water). TEM images were acquired using a by Tecnai G^2^ F30 S-TWIN high resolution transmission electron microscope (ThermoFisher, Eindhoven, Netherlands) operated at 300 kV. The TEM samples were prepared as follows: a small amount of sample was diluted in deionized water. Then 10 µL of the diluted solution it was placed onto a 400-mesh holey carbon coated Cu grid and let it to dry in air prior to the analysis. 

### 2.4. Antimicrobial Evaluation 

Microbial strains were purchased from American Type Culture Collection (ATCC, US). Glycerol stocks were streaked on LB agar to obtain 24 h cultures to be used for all further studies.

#### 2.4.1. Growth Inhibition

To qualitatively assess the antimicrobial potential of obtained nanoparticles, an adapted diffusion method in nutritive agar was performed. We used 4 bacterial strains models, belonging to Gram-positive (*Staphylococcus aureus* ATCC^®^ 23235 and *Enterococcus faecalis* ATCC^®^ 29212) and Gram-negative (Escherichia coli ATCC^®^ 25922 and *Pseudomonas aeruginosa* ATCC^®^ 27853) groups and also a yeast model, *Candida albicans* ATCC 10231. Briefly, 0.5 McFarland (1–3 × 10^8^ CFU (colony forming units)/mL) suspensions in sterile saline were obtained from overnight cultures, previously cultivated in nutritive agar. The obtained microbial suspensions were utilized to swab inoculate Mueller Hinton agar Petri dishes, as for disc diffusion technique described in CLSI 2020 standard (https://clsi.org/, accessed on 1–29 October 2020) [55]. Then, 10 µL of each of the obtained suspensions and controls were drop-added in the inoculated Petri dishes. Plates were incubated for 20 h at 37 °C and the diameter of growth inhibition (mm) was measured.

#### 2.4.2. Minimum Inhibitory Concentration (MIC Assay)

For establishing the MIC (minimum inhibitory concentration) values of the obtained functionalized silver nanoparticles we utilized a microdilution method performed in nutritive broth. The sterile broth was added in sterile 96 well plates and binary dilutions of each tested compound were performed in a final volume of 150 μL. After realizing the binary dilutions, 15 μL of microbial suspension adjusted to an optical density of 0.5 McFarland (1.5 × 10^8^ CFU/mL) were added in each well. The MIC values were established by naked eye analysis and spectrophotometric measurement (Abs 600 nm). Each experiment was performed in triplicate and repeated on at least three separate occasions.

#### 2.4.3. Biofilm Inhibition 

To assess the effect of tested nanomaterials on the biofilm formation of tested microbial strains, experiment was set up in 96 well plates, in a similar fashion as described in MIC evaluation protocol. Microbial cells adjusted at a 10^6^ CFU/mL density were grown in nutrient broth in the presence of various concentrations of the tested nanomaterials. The obtained plates were incubated for 24 h at 37 °C and after incubation the microbial suspension (culture) was gently removed. The obtained biofilms attached on the 96 well plate’s walls were washed with sterile saline buffer and then biofilms were fixed with cold methanol for 5 min. After fixation methanol was removed and air-dried plates were stained with 1% crystal violet solution for 15 min. After staining plates were washed and allowed to dry at room temperature. Biofilm formation ability was assessed by adding 150 µL of 33% acetic acid solution to release the dye included in the biofilm cells. The intensity of the resulting solution (reflecting the intensity of biofilm development) was measured with a spectrophotometer (Abs 490 nm).

## 3. Results and Discussions

### 3.1. UV-VIS Results for Samples Obtaining at the Room Temperature

The optical and structural properties and the stability of the obtained samples were evaluated by UV-VIS spectroscopy. UV-Visible spectroscopy is one of the most widely used techniques for structural characterization of silver nanoparticles [56]. The techniques are sensitive to the surface plasmon excitation of silver nanoparticles which are represented by an absorption peak and evaluate the stability and characteristics of AgNPs [13]. 

**Figure 3:**
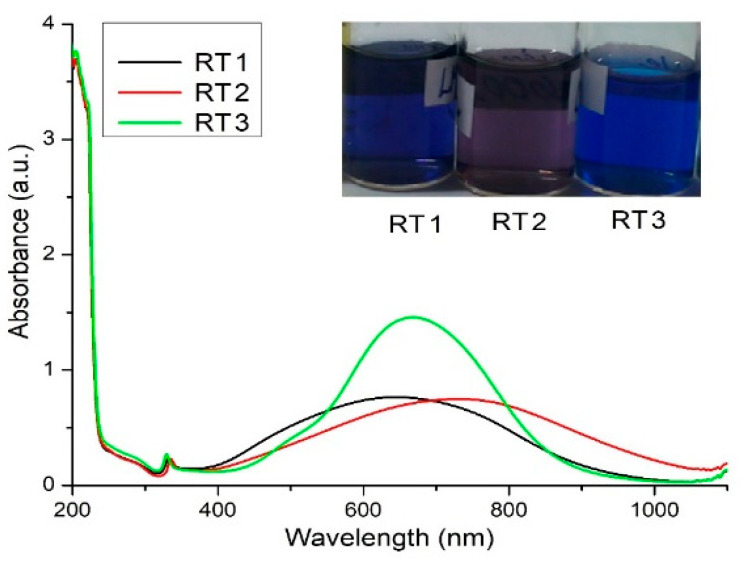
UV-VIS spectra of samples encoded RT.

The obtained silver nanoparticle solutions at room temperature named RT are different in colors (Figure 3) and show that using the various volume of one of the reducing agents (in this case, sodium borohydride), leads to a variety of obtained samples. The blue color of silver nanoparticle solutions indicates the presence of nanoparticle formation differently than the spherical shapes, like rods, triangular, or truncated shapes, and probably manifest the agglomeration tendency in time.

The samples obtained by chemical reduction at room temperature encoded RT show peaks around the value of the wavelength of 320 nm indicating the ions silver presence [57] and peaks around values 600–800 nm indicating the presence of silver nanoparticles with anisotropic shapes. Instead of a single peak about 400–420 nm values, characteristic of spherical particles [49,58] two peaks appeared indicating different shapes than spherical shapes and confirmed by the predominated blue color of the obtained solutions (Figure 3). Various colors of the obtained nanoparticles solutions encoded RT result from the surface plasmon resonance of nanoparticles indicating by their color a certain shapes and sizes. Shifting the plasmon resonances to values of wavelength 600–800 nm (Figure 3) indicating an increasing of silver nanoparticles sizes and modified their shapes to the triangular or bipyramidal shapes [59].

The spectra show a shifting of the absorption band for samples encoded with RT1 and RT2 prepared with the lesser and highest amount of the strong reducing agent (Figure 3).

The sample encoded RT3 synthetized with the medium concentration of the strong reducing agent (NaBH_4_) show a small peak around values 460–490 nm which indicate probably, the presence of some spherical shapes of silver nanoparticles (Supplementary Materials, Table S1). The presence of the next peak formed show a large band around value 600–700 nm indicating an agglomeration tendency of silver nanoparticles synthetized. 

Using highest quantity of the reducing agent for the sample RT2, probably the kinetic of reaction is influenced the core formation during nucleation reaction. The resulted silver nanoparticles in the presence of the capping agent (PVP), drive to the triangular shapes nanoparticles. The PVP agent is acting also as a directing agent, in this case, confirmed by the color sample (violet color) with a slight agglomeration tendency confirmed by UV-VIS data. In this case using various amount of the strong reducing agent, stabilizing agent and silver precursor the shape, size and the stability of the final colloidal solutions are tuned.

Based on UV-VIS data, the chemical reduction proposed by varying the ratio of reduction agents lead to the control of sizes and shapes of nanoparticles. The UV-VIS spectra (Figure 3) indicate the AgNPs presence with different a shapes, sizes and polydispersity confirmed by the color of solutions.

Using the sodium borohydride (NaBH_4_) as reducing agent in the chemical reduction synthesis led to silver nanoparticles with specific sizes and shapes indicated by the color of obtained solution. The blue dark or lighter color is a specific for chemical reduction using sodium borohydride and indicate anisotropic shapes. Pal et al. [60] showed as the truncated shapes proven a better antibacterial efficacy than the spherical nanoparticles shapes because these edges can generate damages of the cell walls. Based on the information obtained from UV-VIS results the obtained nanoparticles might possibly register an antimicrobial activity due to their shape [27] but the polydispersity indicate by the shape of graphic could affect the antimicrobial effect [61,62]

### 3.2. DLS Analysis Data for Obtaining Samples at the Room Temperature

The DLS analysis is mainly used to determine the particle size and distribution in aqueous solutions. The size obtained through DLS analysis is usually larger than TEM analysis, which may be influenced by the agglomeration tendency at nanometric scale [18]. Particles size and surface charge are two main important parameters that can determine the targeting efficacy, deposition, rate of clearance and cellular uptake particles. Dynamic light scattering is the most commonly used method to determining the hydrodynamic diameter of the particles suspended in solution. Particles with zeta potential between −10 mV and 10 mV are considered neutral and prone to aggregation, while particles with 10 mV ≤ │ζ│ ≤ 30 mV are indicating that they can repel each other and assure an incipient agglomeration but, if the │ζ│ increase further, the stability increase considerable and minimal or no aggregation occurs [63]. By DLS, the polydispersity of the particles can be also evaluated. A polydispersity index as PI close to (1) indicates large variations in particle size, while values close to 0 value indicate that the particles are monodispersed [64].

The DLS direct light scattering analyses for both chemical reduction methods bring new information related to the samples as well as reinforced the assumption from UV-VIS data. The zeta potential values of samples obtained by the chemical reduction at room temperature shown a relatively better stability of samples proven by zeta potential (the obtained values are ~30 mV) (Table 3). Thus, for samples encoded with RT obtained by the chemical reduction at room temperature it was registered values of diameters less than 100 nm (Table 3) and Pd index values shown a stability of samples obtained at room temperature (Pd index > 0.2) [63]. Diameters of particles are nearly similar sizes (Table 3) proving that even one of reactants are quantitative modified and keeping same synthesis condition it might obtain constant sizes of silver nanoparticles.

**Figure 4:**
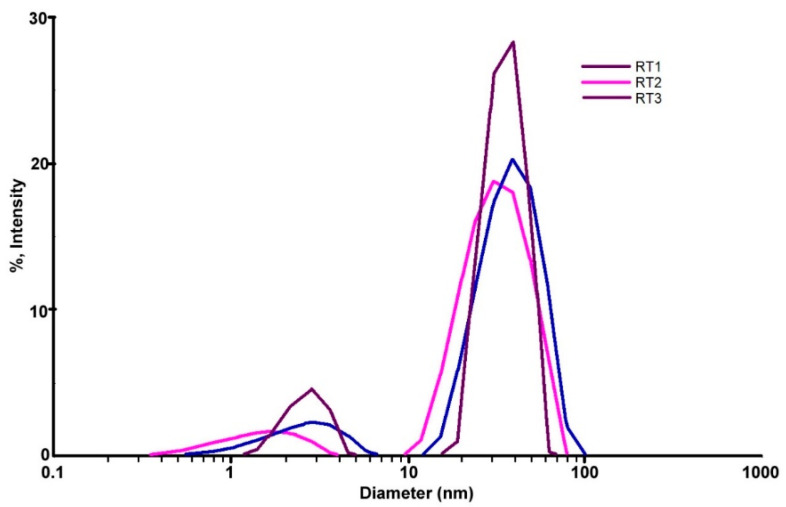
Superposed DLS images for samples obtained by chemical reduction at the room temperature.

The values mentioned in the Table 3 shown the shape and size distribution of nanoparticles are relative homogeneous confirmed, also by UV-VIS data (Figure 3). All samples show a mono-modal distribution of nanoparticles confirmed by obtaining diameter values from DLS test (Figure 4). The samples encoded RT1 and RT2 in the DLS table (Table 3) shown a larger sizes distribution confirmed by the Pd index values. Thus, all samples have a large distribution of sizes and shapes shown an agglomeration tendency. The sample encoded RT3 showed a narrow peak with mono-modal distributions compared with the two samples, probably the volume added of the reducing agent is appropriate to get a monodispersed distribution. These observations are sustained by the UV-VIS graphic (Figure 3) shown a large band width for each samples synthetized. Probably, external factors as ambient temperature, the speed of stirring, time of stirring are one of those components that could influence the final results. It can be noted that the external factors could influence the thermodynamic and kinetics of the synthesis reaction. Using a variation of the strong reducing agent volume can be seen the tendency of nanoparticles to form clusters with average 10 nm sizes.

The DLS image from the sample RT2 shown a relative stability, confirmed by the ζ potential value (−47.53 mV) but smaller value of the polydispersity (Figure 4) comparing with the others values of samples. The value of Pd index indicate a low stability and agglomeration tendency higher comparing with the rest of studied samples. These observations are confirmed by the graphic shape from the UV-VIS (Figure 3), for sample RT2, which show a large area with small peak values indicated a large polydispersity of sizes nanoparticles. The smaller volume of the strong reducing agent (NaBH_4_) (sample coded with RT2) driven to the heterogeneity of size distribution (Figure 4). Comparing DLS graphics for the obtained sample (Figure 4) it is noteworthy that relative monodispersed sizes of nanoparticles can be obtain at the right volume of the strong reducing agent. Probably, small volume of the strong reducing agent led to the obtain nanoparticle with high polydispersity which drive to a rapid agglomeration of silver nanoparticles. The synthesized samples with a similar volume of the strong reducing agent could drive at samples with comparable properties but having particularities depending on the external factors during synthesis.

**Figure 5:**
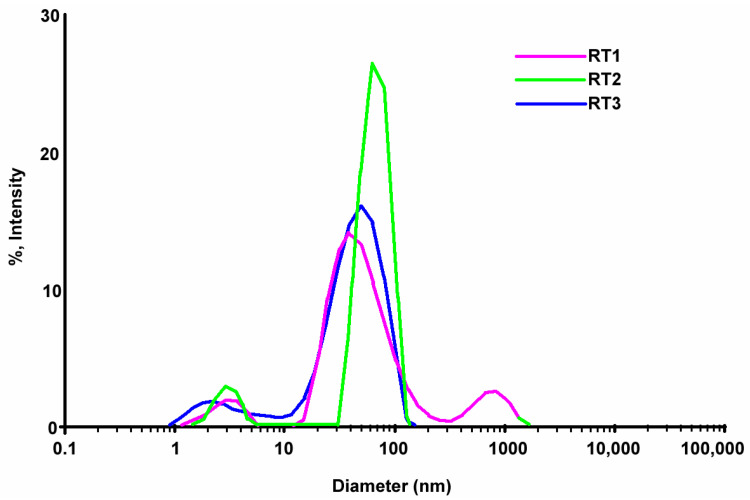
DLS images results after 8 months.

According to the DLS obtained results after 8 months from the first test it can be seen all samples have multimodal dispersity (Figure 5). All samples show a slight tendency of NPs agglomeration. The sample coded with RT3 registered a higher peak comparing with the sample RT1 and RT2. The encoded sample RT3 showed a diminished quantity of nanoparticles formed with size less than 10 nm, but we noticed an increasing nanoparticle with sizes over than 50 nm. The graphics show a multimodal distribution less the sample RT3 (Figure 5). Samples RT1 and RT2 show a higher agglomeration of particles with size higher than 50 nm. Probably, a higher or lesser added quantity of the reducing agent lead in time, to the various morphologies and sizes with agglomeration tendency. According to the information received from DLS results the polydispersity of the obtained nanoparticles could affect the antimicrobial activity [27,61,62].

### 3.3. TEM Analysis Data for Obtaining Samples at the Room Temperature

**Figure 6:**
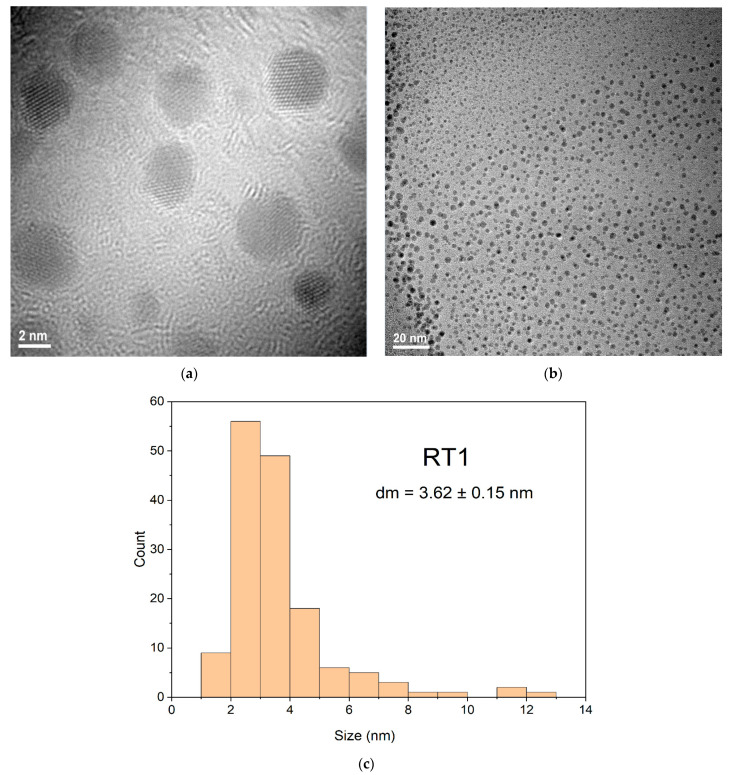
TEM images of AgNPs sample coded RT1 at two different magnifications (**a**,**b**) and the appropriate histogram (**c**).

TEM results for samples RT1 and RT3 show a relative homogeneous distribution confirmed by corresponding DLS data (Figure 4). The spherical shape combined with the truncated shapes shown in TEM images (Figure 6) of the sample named RT1 are sustained both by the color of obtained solution and the shape of UV-VIS graphic results (Figure 3). The UV-VIS shape graphic (Figure 3) corresponding to the sample encoded RT3 is very large and confirm the polydispersity sizes and different shapes obtained.

Based on TEM results from RT1 and RT3 particle sizes were measured with Image J software. The obtained values were plotted using OriginPro2021 and statistically evaluated for average size and standard deviation measurements. From the obtained values the error was calculated. Thus, for these measurements, 250 nanoparticles from different areas were taken into consideration, for each sample.

TEM images represented at different resolutions, for both samples coded RT1 and RT3, show different shapes of silver nanoparticles obtained. Comparing shapes of silver nanoparticles of samples RT1 and RT3 shown that a larger volume of the strong reducing agent (NaBH_4_) added at the sample RT1 could influence the kinetic reaction for nanoparticles formation led to obtaining oval or spheres shapes (Figure 6a,b) and it is noticed that no agglomeration tendency for the particles is observed. For sample encoded RT1 probably, the larger volume added of the strong reducing agent influence both the rapid Ag^0^ nuclei formation drives to obtaining of small nanoparticles (Figure 6b). Thus, for RT1 sample (Figure 6c) show mainly nanoparticles formation with sizes less than 4 nm with a bi-modal distribution (if considering the few particles larger than 10 nm) (Figure 6c).

**Figure 7:**
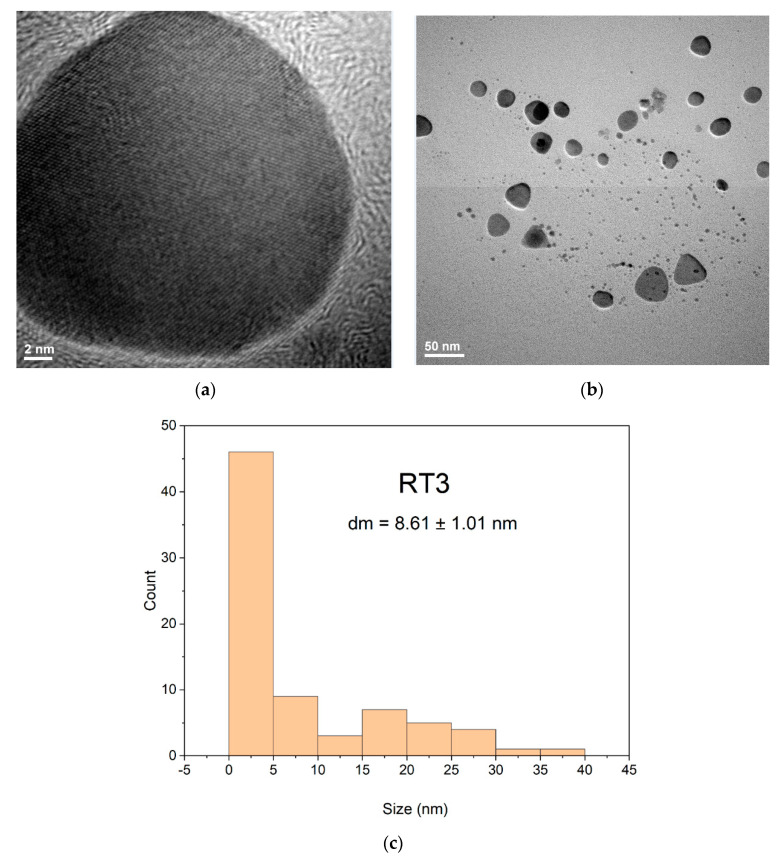
TEM images for AgNPs sample coded RT3 at two different magnifications (**a**,**b**) and the appropriate histogram (**c**).

The RT3 sample show a polyhedral or oval shapes of nanoparticles (Figure 7b) and presents a monomodal distribution, but with a maximum particle sizes in the area of 1–5 nm more [18,28,46], with slightly agglomeration tendency (Figure 7a,b). The sample RT3, according to the TEM, shows a bimodal distribution of the nanoparticles. The smaller fraction of the AgNPs have the average diameter of 8.61 nm being and a larger fraction which is usually bellow 40 nm. The morphologies for both samples can sustain the presumption that both samples RT1 and RT3 have antimicrobial activity, depending on their size and shape and agglomeration tendency. TEM images confirmed results obtained at UV-VIS and DLS for both analyzed samples. DLS images indicate different sizes of nanoparticles formation mainly for both samples coded RT1 and RT3 with limited agglomeration tendency (Figure 4) depending the reaction conditions.

**Figure 8:**
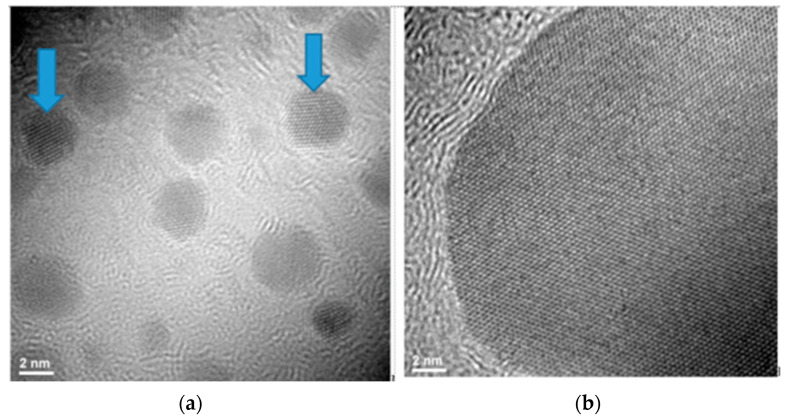
Structure of nanoparticles with twinned planes formation for samples RT1 (**a**) and RT3 (**b**).

TEM images show for both samples coded RT1 and RT3 a slight difference comparing their structures. The sample coded RT1 presented a multiply twinned planes (Figure 8a) comparing with sample coded with RT3 (Figure 8b) with uniform twinned planes. This information related the structure of nanoparticles formed could influence the antimicrobial activity. Those nanoparticles presented multiply twinned planes could record better antimicrobial activity than nanoparticles with uniform twinned planes [14,32,35,65,66]. Both samples show a bi-modal or mono-modal distribution of nanoparticles and confirm by DLS and UV-VIS test results an agglomeration tendency [31,39,67,68].

### 3.4. UV-VIS Results for Samples Obtaining by the Solvothermal Method

UV-VIS analysis was done with samples solution diluted at the concentration 10% or 15%. Due to the high viscosity and dark brown color of the obtained samples all solutions were diluted in order to allow the transmission of light. The resulting samples named HT (solvothermal synthesis at high temperature) have different colors from deep yellow color to dark brown depending on the temperature and time applied during the solvothermal synthesis.

**Figure 9:**
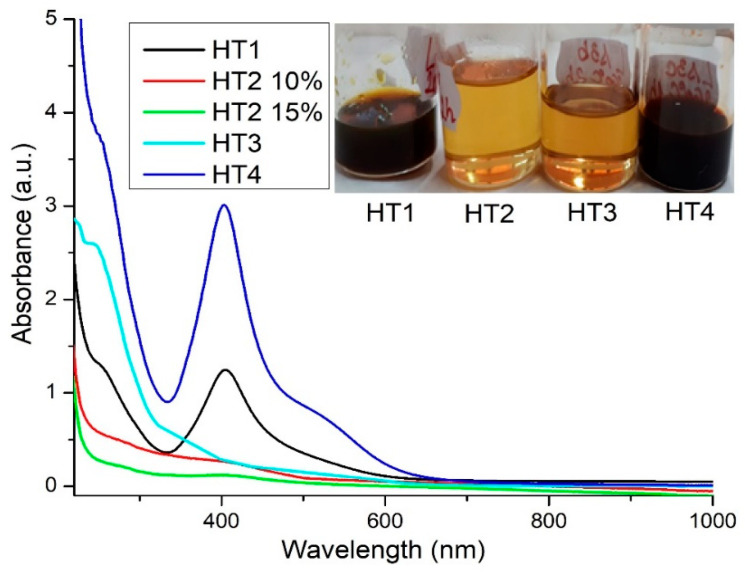
UV-VIS spectra of solvothermal synthesis with PEG and PVP.

UV-VIS spectra (Figure 9) for the solvothermal synthesis with polyethylene glycol used as the capping agent and solvent and PVP as the capping agent at different temperature and time, highlights the formation of AgNPs for the samples used at 200 °C during 4 h and 260 °C, 1 h, pressure-3 bar. The samples obtained after solvothermal synthesis have the deep yellow color to dark brown color (Figure 9).

UV-VIS spectra (Figure 9) for all samples encoded with the name HT1, HT2, HT3, HT4 indicated the presence of nanoparticles highlighted by peaks around 400–410 nm. The samples coded with HT1 and HT4 shown the presence of nanoparticles formation comparative the broad spectra of the sample coded with HT1 is the largest than for sample HT4. The absorbance corresponding to the wavelength around 403–407 nm (Supplementary Materials, Table S2) indicated for both samples having a yellow component color probably, spherical shapes formation. The samples encoded HT1 and HT4 have dark color (Figure 9) due to the concentration of solution obtained during the solvothermal processing. Both samples are synthesized at different temperatures and times. The UV-VIS results showing that at temperatures 200 and 260 °C under the boiling temperature of PEG the silver nanoparticles can be synthesized in certain shapes and sizes of nanoparticles. The shape of the spectra (Figure 9) for both samples HT1 and HT4 and values for the absorbance showed the maximum value of wavelengths at 400–410 nm. The height and sharpness of graphics confirm that silver nanoparticles have, probably, a spherical shape, with relative low polydispersity of nanoparticles sizes for both samples coded with HT1 and HT4. The other two samples encoded HT2 and HT3 at the same temperatures 200 °C respectively 260 °C but different time of reaction, the UV-VIS results indicate no silver nanoparticle formation. Probably, reaction conditions are not propitious to synthetized nanoparticles.

### 3.5. DLS Analysis Data for the Obtained Samples

**Figure 10:**
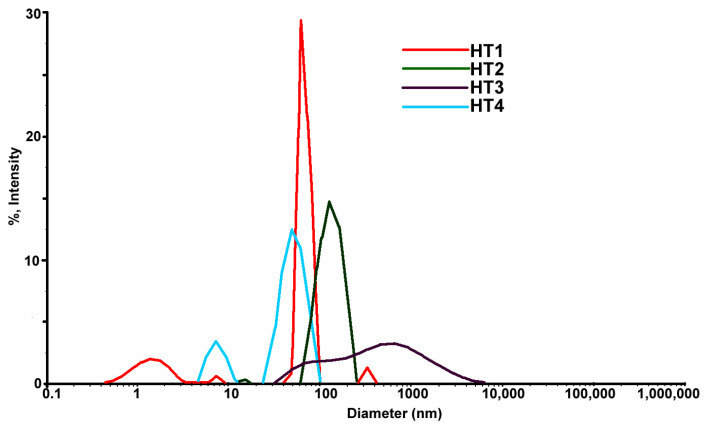
Superposed DLS images for samples coded HT obtained by the solvothermal method.

DLS analysis were done in special cuvettes with diluted solution. The DLS images from samples obtained by solvothermal method (Figure 10) show by comparison with samples obtained at the room temperature (Figure 3) an agglomeration tendency with the main component average 50 nm of sizes (Figure 10). The table (Table 4) with values of the medium diameter of formed nanoparticles at high temperature confirmed that sizes are higher compared with nanoparticles formed at room temperature (RT) and present less stability indicated by ζ values registered > −30 mV.

The graphic images from Figure 10 show that nanoparticles synthetized by the solvothermal method have big sizes (average sizes 25–50 nm sizes) comparing with nanoparticles synthetized at room temperature Figure 4. AgNPs obtained at room temperature presenting a relatively less polydispersity than solutions obtained at the high temperature. Graphic from the figure (Figure 10) indicates that samples have multi modal distribution sizes. Sample coded HT1 has the nearest size distribution probably the temperature and time are adequate to obtain nanoparticles with controlled sizes less 100 nm. The sample coded HT2 was treated at the same temperature as the HT1 sample but with a longer time during synthesis. Probably a long time could affect the size stability of nanoparticles and presented an agglomeration tendency. Comparing samples HT3 and HT4 processed at the same temperature and different times of synthesis it is noticed that samples HT3 with longer time of synthesis than HT4 lead to obtain a large nanoparticle, a multi modal size. These observations are sustained by UV-VIS data (Figure 9) show the presence of nanoparticles formation only for samples coded HT1 and HT4 Comparing graphics and data obtained by DLS tests between these two syntheses it can be seen that samples obtained at room temperature (Figure 4) show higher stability prove by ζ potential than solvothermal obtained solutions and sizes of nanoparticles less than 50 nm (Figure 10). Solvothermal method show a relatively less stability of the obtained solutions and size of nanoparticles higher comparing with RT samples.

**Figure 11:**
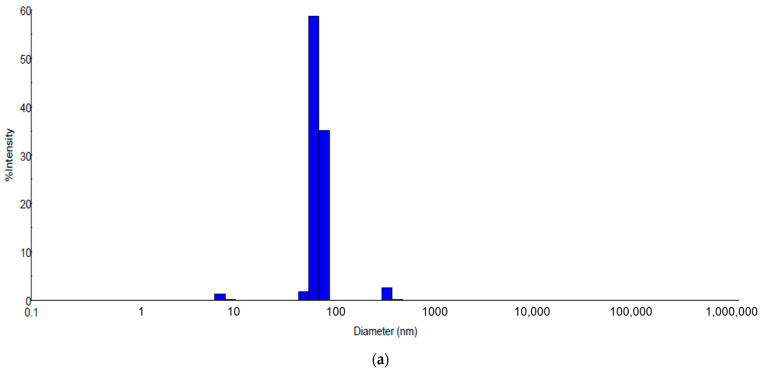

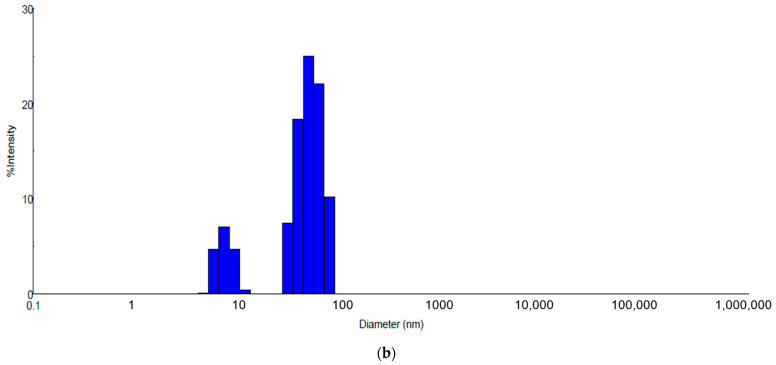
DLS images for samples named HT1 (**a**) and HT4 (**b**) obtained by solvothermal method, after 8 months.

DLS graphic (Figure 11) of samples obtained by the solvothermal method after 8 months show a stabilization of solutions comparing with silver nanoparticles obtained at room temperature (Figure 5) for the same time. Probably, the temperature during the synthesis reaction influenced the growth of the nanoparticles [13]. The DLS graphics (Figure 11) show a sizes stabilization around value of 50 nm and a reduction of polydispersity comparing than DLS images of the same samples tests before (Figure 10).

### 3.6. TEM Results of Samples Obtained by the Solvothermal Method

Based on TEM results from HT1 and HT4 particle sizes were measured with Image J software. The obtained values were plotted using OriginPro2021 and statistically evaluated for average size and standard deviation measurements. From the obtained values the error was calculated. Thus, for these measurements, 250 nanoparticles from different areas were taken into consideration, for each sample.

**Figure 12:**
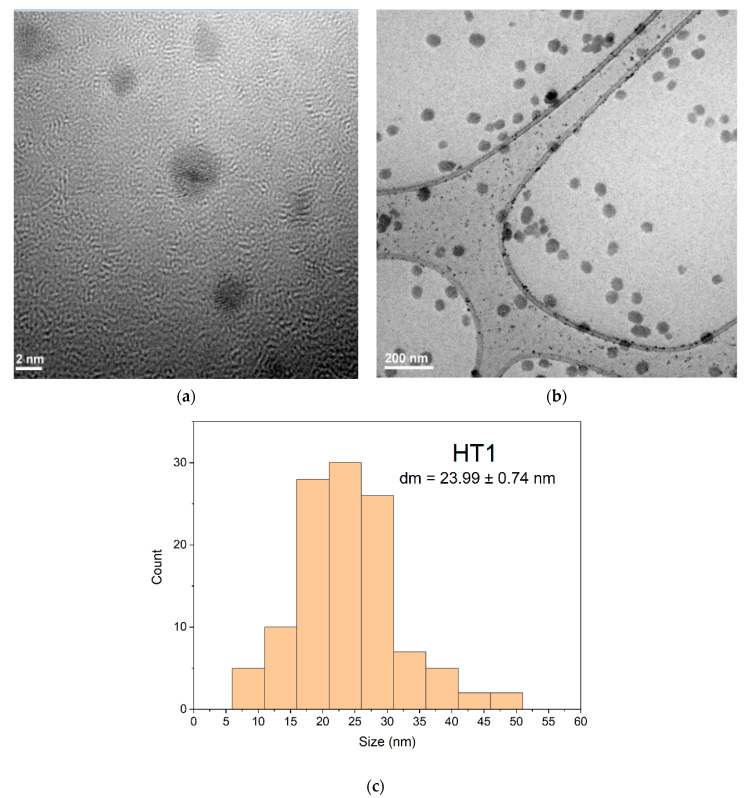
TEM images for the AgNPs sample HT1 at two different magnifications (**a**)—high magnification and (**b**)—low magnification) and the appropriate histogram (**c**).

TEM images of samples obtained by the solvothermal method encoded with HT1 and HT4 at different resolutions showed some morphological differences in the obtained nanoparticles. The AgNPs sample coded HT1 treated at 200 °C (Figure 12) show a uniform spherical shapes formation. The morphology of sample coded HT1 (Figure 12a,b) indicate a relative uniform dispersity of sizes and predominant spheres shapes formation. The TEM for sample HT1 (Figure 12a) indicate at the scale of 2 nm the presence of multiply twinned plane. The histogram corresponding to the sample HT1 (Figure 12c) show a bimodal distribution of nanoparticles sizes with main diameter size around 24 nm value. Nanoparticles formed presents an agglomeration tendency proven by size of nanoparticles more than 50 nm (Figure 12c).

**Figure 13:**
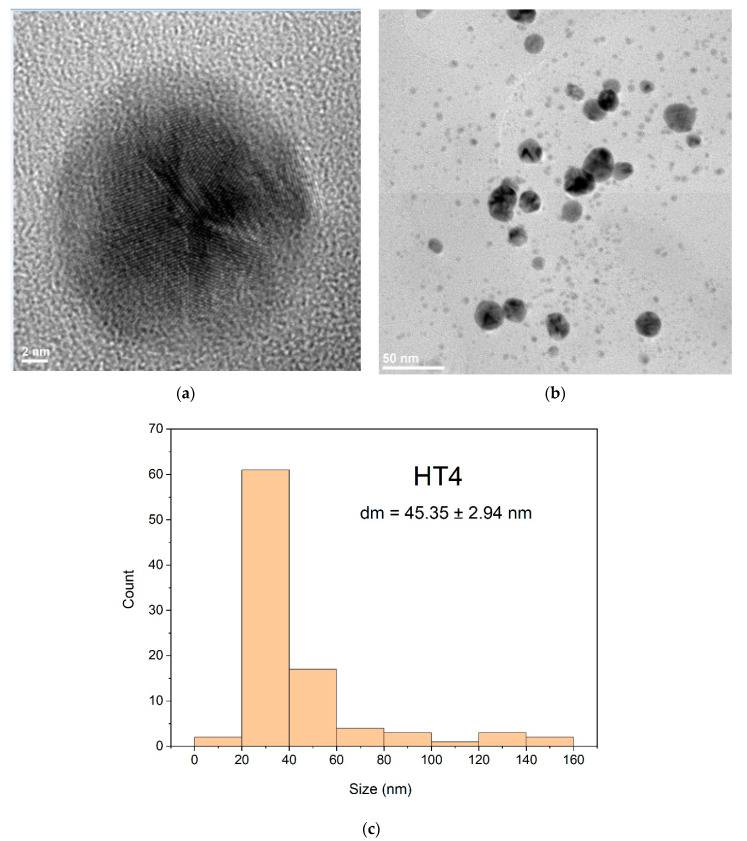
TEM images for the AgNPs sample HT4 at two different magnifications (**a**)—high magnification and (**b**)—low magnification) and the appropriate histogram (**c**).

The sample HT4 (Figure 13b) indicate spheres particles formation with agglomeration tendency formed nanoparticles bigger size around 45 nm diameter, than HT1 (Figure 13c). The distribution of the AgNPs for samples HT1 and HT4 indicated by TEM images (Figure 12b and Figure 13b) are sustained also by obtained DLS results [69] (Figure 10). The structure of nanoparticles for HT1 and HT4 samples indicate in TEM image (Figure 12a and Figure 13a) formation of multiply twinned planes developed on multiply direction comparing samples obtained by the room temperature method with twinned plans (Figure 8a,b).

Analyzing diameters distribution for samples encoded HT1(Figure 12c) and HT4 (Figure 13c) based on TEM results confirm the observation from DLS images (Figure 10) that the sizes of nanoparticles have a bi-modal distribution. Both samples formed nanoparticles with sizes average sizes around values 23–45 nm comparing with samples obtained at the room temperature which are less than 10 nm (Figure 6c and Figure 7c). Probably, the temperature and time of during synthesis drive to a uniform morphology but sizes of nanoparticles and agglomeration tendency are higher comparing with samples obtained at room temperature. Different syntheses routes to obtain AgNPs with antimicrobial activity lead to various results depending the reaction condition [61].

## 4. Antimicrobial Activity

**Figure 14:**
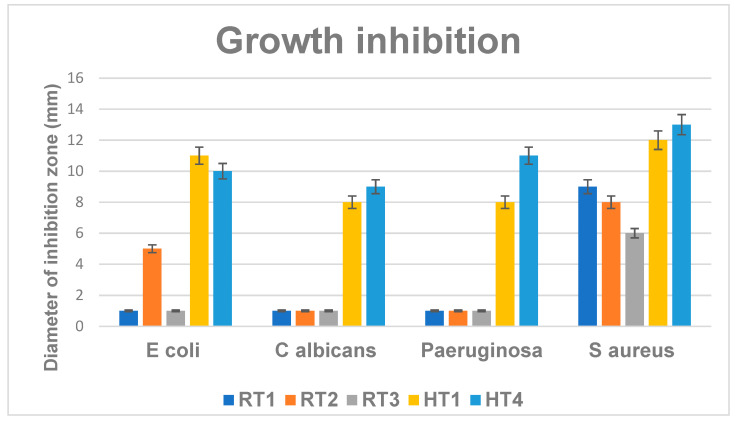
Graphic representation of growth inhibition zones (mm) developed after 24 h of incubation of microbial strains in the presence of the obtained NPs.

The antimicrobial activity of the developed NPs was extensively investigated by qualitative (growth inhibition diameter) and quantitative (MIC) experiments in both planktonic and biofilm cultures [14,28,32,39,70,71]. From the synthetized samples were selected for antimicrobial tests samples encoded RT and from solvothermal route HT1 and HT4 since they showed uniform nanostructured particles [13,72,73]. The most significant antibacterial activity expressed for all of the evaluated strains was obtained for the samples coded with HT1 and HT4 obtained by solvothermal synthesis (Figure 14). The antimicrobial effect of the evaluated nanoparticles was obtained against both for the Gram positive species (*S. aureus)* and Gram negative (*P. aeruginosa, E. coli*) bacteria, but also against the opportunistic yeast *C. albicans*. HT1NPs and HT4NPs provided diameter higher diameter of inhibition zones, ranging 8–12 nm in all the test situations (Figure 14). Comparing these qualitative results among the two types of samples (RT and HT), the results suggest that HT NPs show enhanced antimicrobial effect, as compared to RT samples.

HT samples showed spherical shapes of nanoparticles formation indicated by the TEM images (Figure 12 and Figure 13) and have a PEG-based surface which, along with the size and shape strongly influence the antimicrobial activity. According to DLS results at the sample HT4, even if nanoparticles sizes are over 100 nm the relative homogeneity of sizes, their morphology (with multiply twinned planes formed) and concentration of the solution helps to improve its efficacy.

Comparing the RT samples with samples obtained by solvothermal synthesis (HT) ones it is noticed that RT samples obtained by chemical reduction at room temperature show better efficiency relatively better against gram-positive bacteria *S. aureus* as compared to their antimicrobial effect on the other microbial strains. However, solvothermal samples (HT) clearly show enhanced antibacterial effect against *S. aureus*, with average diameter of inhibition zones of 11.5 cm, while growth inhibition diameter averages 7 cm for RT samples in this Gram-positive bacteria.

It is noticeable that the samples delivered from the synthesis at the room temperature has the efficiency for Gram-positive bacteria. This is might be possible the less concentration of AgNPs could affect the antimicrobial efficiency for the Gram –negative bacteria while for Gram-positive bacteria the silver ions (Ag^+^) could bind to active sites of the *S. aureus* disrupting the bacteria cell by thiol-redox homeostasis and increased ROS production, which induced bacteria death finally [29,44].

**Figure 15:**
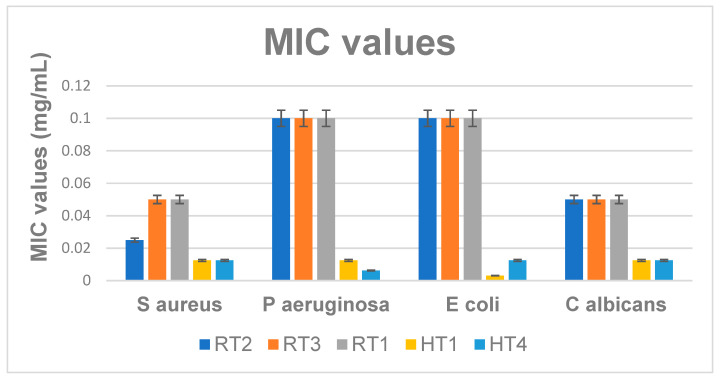
Graphic representation of the MIC values (mg/mL) for the evaluated samples Obtained after 24 h of incubation of microbial strains in the presence of the obtained NPs, *p* < 0.05 when comparing MICs of RT samples to MICs of HT samples.

The minimum inhibitory concentration (MIC) is a quantitative method, based on obtaining a serial binary microdilution in liquid nutritive media. The graphic in Figure 15 show lower MIC values for all the tested strains, in the presence of AgNPs obtained by solvothermal synthesis (encoded HT). These results are also consistent with the qualitative assay, measuring the growth inhibition zone. While the MIC values obtained for HT samples are lower than 0.01 mg/mL, the MICs obtained for RT samples range 0.025–0.1 mg/mL (Figure 15). In general, the better antimicrobial activity for the HT series can be associated also with the presence of PEG which, can improve the internalization of these AgNPs, even if these nanoparticles are larger and spherical comparing with the RT series (smaller and sometime truncated triangular, both characteristics being reported in the literature as favoring the antimicrobial activity) [74].

**Figure 16:**
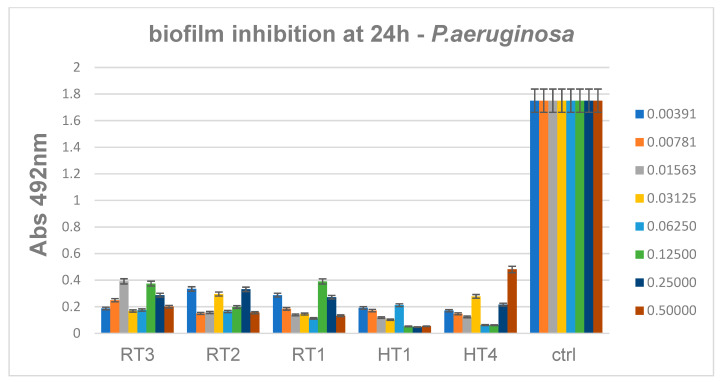
Inhibition of *P. aeruginosa* biofilm development in the presence of the obtained AgNPs for 24 h, *p* < 0.001 when comparing Abs 492 nm values of RT and HT samples control.

The antibiofilm activity results proved that the NPs obtained by solvothermal method and encoded HT possess the better inhibitory properties against *P. aeruginosa* strain (Figure 16). These results are consistent to previous antimicrobial assay, suggesting solvothermal obtained AgNPs have a higher antibacterial activity, however it seems that biofilm inhibition is relatively constant among the solvothermal samples synthetized at different temperatures. (Figure 16) reveals that minimum biofilm inhibitory concentrations obtained for all HT samples range 0.06–0.12 mg/mL.

These data show that biofilms are more resistant to nanoparticles, since the minimum concentrations necessary for significant biofilm inhibition are at least two times higher, as compare to the minimum inhibitory concentrations of free-floating (planktonic cells). Lowest MIC values obtained for HT samples ranging 0.03–0.06 mg/mL, as revealed by figure (Figure 15). For solvothermal synthesis samples the efficiency may be due to the high concentration, PEG contain of samples, shapes and sizes of nanoparticles. For samples obtained at room temperature shapes, sizes and polydispersity of silver nanoparticles probably improve the efficiency against gram negative as *P. aeruginosa* and Gram-positive *S. aureus* bacteria [35,50].

**Figure 17:**
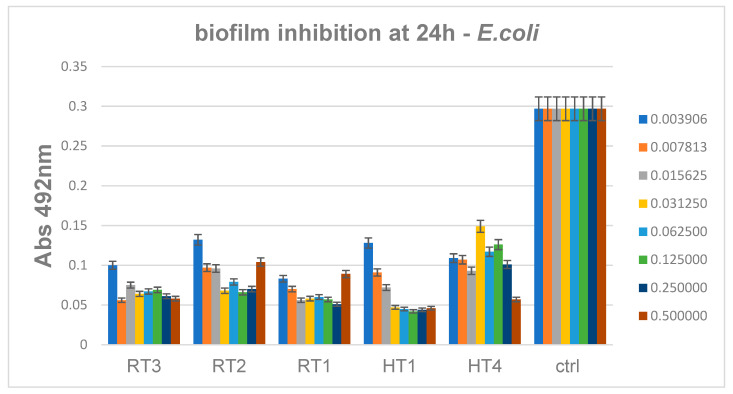
Inhibition of *E. coli* biofilm development in the presence of the obtained AgNPs for 24 h, *p* < 0.001 when comparing Abs 492 nm values of RT and HT samples to control.

For the Gram-negative evaluated species, *E. coli*, results showed that biofilms are slightly more efficiently inhibited by the solvothermal nanoparticles encoded HT, as compared to the other samples (Figure 17). For these samples the minimum concentrations required for biofilm inhibition range 0.06–0.12 mg/mL, similar to *P. aeruginosa* biofilm inhibition results.

**Figure 18:**
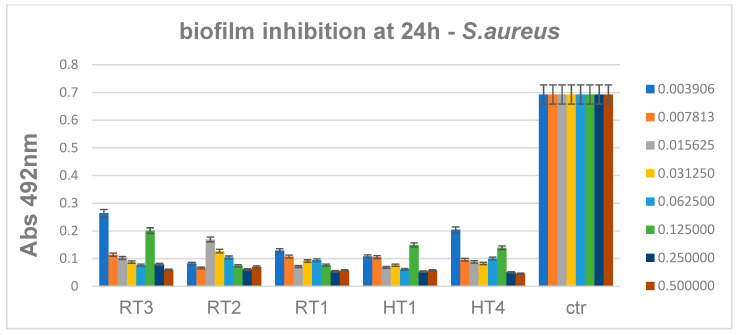
Inhibition of *S. aureus* biofilm development in the presence of the obtained AgNPs for 24 h, *p* < 0.001 when comparing Abs 492 nm values of RT and HT samples to control.

*S. aureus* biofilm development was constantly inhibited by all of the obtained AgNPs, when they were used in concentrations higher than 0.12 mg/mL (Figure 18). These results suggest that the Gram-positive biofilms may be more resistant to the activity of AgNPs regardless their synthesis route.

**Figure 19:**
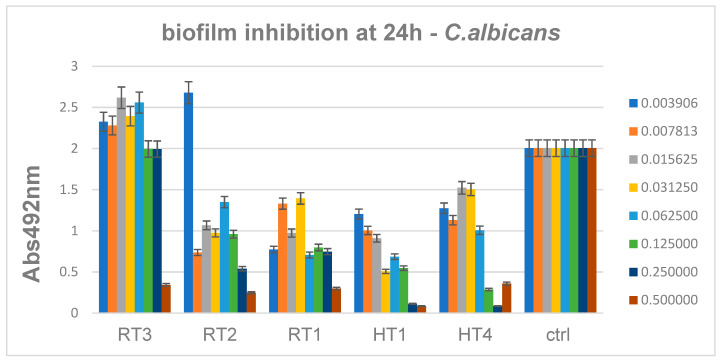
Inhibition of *C. albicans* biofilm development in the presence of the obtained AgNPs for 24 h.

Antibiofilm data showed also that *C. albicans* monospecific biofilms are inhibited mainly by AgNPs obtained by solvothermal method at high concentration of AgNPs. The minimum biofilm inhibitory concentrations of samples encoded HT1-HT4 range 0.06–0.12 mg/mL, similar to the results obtained for Gram negative bacteria, while the biofilm inhibitory concentrations obtained for the RT1 sample are slightly higher ranging 0.12–0.25 mg/mL (Figure 19). For *C. albicans* results are relatively accepted for high concentration and for solvothermal samples.

## 5. Conclusions

This work aimed to obtain antimicrobial AgNPs, by using two synthesis approaches, namely chemical reduction at room temperature (RT) and solvothermal method at high temperature (HT). The results showed that AgNPs obtained by the room temperature (RT) method registered a various morphology with truncated or bipyramidal aspect and the sizes around 1–20 nm. Samples obtained at HT with spherical shapes and size of nanoparticles more than 50 nm have relatively better antimicrobial activity than AgNPs coded with RT. Also, these present a lower agglomeration tendency than the nanoparticles obtained at the room temperature. These differences could be related to the condition of the medium reaction utilized during synthesis which driven to a variation of shapes and sizes. AgNPs obtained at room temperature are the easiest method for obtaining nanoparticles but, shapes, sizes and stability in time are highly influenced by environmental conditions. The solvothermal method is a more expensive method that requires careful monitoring of the reaction that takes place in special conditions. Due to the special conditions in which the reaction takes place, at constant pressure, and high temperatures the AgNPs obtained have sizes range higher compared with the chemical reduction at room temperature, and are more stable and have uniform shapes than the samples obtained at the room temperature. Even if the synthesis at room temperature is easier to achieve, the variables during reaction can lead to different morphologies of nanoparticles and greater tendency towards agglomeration. The antimicrobial activity of the developed NPs depends on the method type, chemicals and synthesis conditions. Samples obtained at high temperature method showed the highest inhibitory effects, being active on a wide range of Gram positive, Gram negative, and yeast strains, which are relevant for human pathology. The obtained AgNPs showed low minimum growth inhibitory concentrations and biofilm inhibitory concentrations, usually ranging 0.03–0.12 mg/mL, values being higher for biofilms, as expected. Comparing both syntheses The results of both syntheses proved once again the significance of the followed method depending on the specific reaction parameters which influence the final morphology of nanoparticles, the physico-chemical and biological properties of the nanoparticles of the noble metals.

## Figures and Tables

**Table 1:** The order and molar concentration of components added for each sample obtained by the chemical reduction at the room temperature method.

Reactants/Molar Concentration (M)		Sample1 (RT1)	Sample 2 (RT2)	Sample 3 (RT3)
sodium borohydride	NaBH_4_	0.06	0.5	0.1
trisodium citrate	Na_3_C_6_H_5_O_7_	0.0299	0.0299	0.0299
silver nitrate	AgNO_3_	0.1 × 10^−4^	0.1 × 10^−4^	0.1 × 10^−4^
polyvynilpirrolidone	PVP K 40	0.7 × 10^−5^	0.7 × 10^−5^	0.7 × 10^−5^
hydrogen peroxide	H_2_O_2_	0.1	0.1	0.1

**Table 2:** Components and conditions of solvothermal synthesis.

Samples Components	HT1	HT2	HT3	HT4
condition of reaction	T 200 °C, 1 h, *p* = 1 bar	T 200 °C, 4 h, *p* = 1 bar	T 260 °C, 2 h, *p* = 1 bar	T 260 °C, 1 h, *p* = 3 bar
PEG 400-888 mg				
PVP K30-80 mL				
AgNO_3_-2 mL				

**Table 3:** DLS results for samples obtained at the room temperature (RT).

Samples	Test Results DLS Model: DELSAMAX PRO		
	Ζ [mv]	Pd Index	Diameter [nm]
RT1	−31.61	0.42	22.7
RT2	−47.53	0.18	22
RT3	−37.2	0.08	19.1

**Table 4:** DLS values for obtaining samples by solvothermal synthesis coded HT.

Samples	Test Results DLS Model: DELSAMAX PRO		
	Ζ [mv]	Ζ [mv]	Ζ [mv]
HT1	−24.92	0.02	68.6
HT2	−9.34	0.08	125.3
HT3	−10.65	0.19	49.8
HT4	−28.29	0.04	27

## Data Availability

Not applicable.

## Supplementary Materials

The following supporting information can be downloaded at: https://www.mdpi.com/article/10.3390/ijms23115982/s1.
