# Supplementary material for: Comparative Antimicrobial Activity of Silver Nanoparticles Obtained by Wet Chemical Reduction and Solvothermal Methods
Source: Int J Mol Sci. 2022 May 26;23(11):5982. doi: 10.3390/ijms23115982 (PMC9180968; doi:10.3390/ijms23115982)
Supplement: Supplementary file 1 [file ijms-23-05982-s001.zip › ijms-1704140-supplementary.pdf]

Comparative antimicrobial activity of silver nanoparticles obtained by wet chemical reduction and solvothermal methods

Liliana Marinescu[1], Denisa Ficai[1], Anton Ficai[1,3], Ovidiu Oprea[1], Adrian Ionut Nicoara[1], Bogdan Stefan Vasile[1], Laura Boanta[2], Alexandru Marin[2], Ecaterina Andronescu[1,3,*], Alina-Maria Holban[4]

[1.] POLITEHNICA University of Bucharest, Faculty of Applied Chemistry and Material Science, Gh Polizu street 1-7, 011061 Bucharest, Romania (*ecaterina.andronescu@upb.ro)
[2.] POLITEHNICA University of Bucharest, Faculty of Power Engineering, Department of Hydraulics, Hydraulic Machinery and Environmental Engineering, 313 Splaiul Independentei, District 6, 060042-Bucharest, Romania
[3.] Academy of Romanian Scientists, Ilfov st 3, 050054 Bucharest, Romania
[4.] University of Bucharest, Faculty of Biology, Microbiology Immunology Department,1-3 Portocalelor Lane, District 5, 77206 Bucharest, Romania

Suplementary materials

Table S1. Intensity values of peaks for samples encoded RT obtained at the room temperature.

**Batch**	**RT1**								
Cycle01	nm	332	638	650	1034	1059	1068	1074	1087
Peaks	A	**0.22699**	**0.76556**	**0.76558**	**0.03492**	**0.04458**	**0.04748**	**0.05371**	**0.08348**
**Batch**	**RT2**								
Cycle01	nm	335	719	727	733	744	1058	1068	1087
Peaks	A	**0.2312**	**0.74844**	**0.74935**	**0.74974**	**0.74845**	**0.14654**	**0.14244**	**0.15822**
**Batch**	**RT3**								
Cycle01	nm	223	333	459	491	496	500	505	659
Peaks	A	**4.31756**	**0.96292**	**0.94999**	**0.90647**	**0.90097**	**0.8989**	**0.89968**	**1.65273**

Table S2. Intensity values of peaks for samples encoded HT obtained by solvothermal synthesis

**Batch**	**HT1**								
Cycle01	nm	407	560	565	573	580	584	587	589
**Peaks**	**A**	**2.2017**	**0.0805**	**0.075**	**0.0674**	**0.062**	**0.0581**	**0.0561**	**0.0556**
Batch	S2a dil 10%; 15%								
**Batch**	**HT2**								
Cycle01	nm	364	367	437	440	444	448	453	457
**Peaks**	**A**	**0.1331**	**0.1267**	**0.0364**	**0.034**	**0.032**	**0.0292**	**0.0272**	**0.0258**
**Batch**	**HT3**								
Cycle01	nm	481	490	493	496	499	506	511	515
**Peaks**	**A**	**0.0444**	**0.0399**	**0.0384**	**0.0374**	**0.0352**	**0.0326**	**0.0306**	**0.0295**
**Batch**	**HT4**								
Cycle01	nm	403	707	710	719	1075	1083	1087	1096
**Peaks**	**A**	**3.0092**	**0.0504**	**0.0487**	**0.0448**	**0.0474**	**0.0638**	**0.0859**	**0.1256**
